# Paracentesis Thoracis

**Published:** 1855-11

**Authors:** W. F. Westmoreland

**Affiliations:** Professor of Clinical Surgery in the Faculty of Paris


ARTICLE V.—*Paracentesis Thoracis.* (Translated by W. F. WESTMORELAND, M. D., from the "Elemens de Pathologie Chirurgical, published in 1854 by M. NELATON, Professor of Clinical Surgery in the Faculty of Paris. Vol. III, p. 459.)

This operation, which for a long time has been designated *empyeme*, is now most frequently known under the name of *thoracentese.* The operation is performed to evacuate liquids accumulated in the plural sacs.

M. LACAZE DUTHIERS has written a most excellent thesis* upon this subject, which may be consulted with very great advantage. In this important treatise we have in *resume* the state of the science upon this subject.

Whatever may be the nature of the liquid accumulated in the chest, it is evident that the effusion may result in death: firstly, by compression of the lung and asphyxia; secondly, by putrid intoxication; thirdly, by gradual exhaustion dependent upon prolonged suppuration; fourth, certain subjects die suddenly without any one being able to give a satisfactory explanation of the cause of such sudden death. Knowing that such accidents may occur, the indication is when they can be foreseen, to give exit to the liquid. The cures in this way, already mentioned by the ancients, have been too frequently repeated within the past few years for there to rest one doubt upon the subject. Paracentesis thoracis is then an operation perfectly rational, and one that we hope will soon occupy the place that it deserves among the theraputical agents of medicine and surgery.

But objections have been made to paracentesis thoracis which at first would appear not without value:

1. *The introduction of air into the Pleural Cavity.*—The introduction of air into the pleural cavity constitutes truly a grave accident, but happily it occurs but rarely in the operation. If this objection has heretofore had some foundation, thanks to the perfection in the mode of operating, it has none at present. Besides, this objection has been observed more particularly in the operation by incision—a very defective method, and one very nearly abandoned.

* These de Paris, No. 14.

2. *The impossibility to empty entirely the Chest.*—The thoracic walls, sustained by the costal arches, have but a limited movement of retreat; on the other hand, the lung cannot distend itself, as it is compressed, and often bound down by false membranes. We would remark, however, that this objection does not hold good in every case. If, for example, we have a case of acute pleurisy, with alarming asphyxia, the lung in such a case has not been so long compressed that its distension would be difficult; the same of acute hydrothorax and recent effusions of blood.

Let us examine by what mechanism the evacuation of the liquid is obtained. As soon as the puncture is made into the chest a certain amount of liquid is discharged immediately, the distended thoracic walls little by little resume their place, and the diaphragm, depressed, assumes its normal position. When this result has been obtained, the cavity of the chest is enlarged at each inspiration, the air penetrating the lung, even if there be a certain amount of liquid still in the cavity of the chest. During expiration, on the contrary, the walls of the chest become depressed, compresses the lung, already distended with air, and while the air escapes through the trachea, a new quantity of liquid passes through the canula; the same phenomena is produced at each respiratory movement until the lung fills the cavity of the chest. This objection is more particularly applicable in chronic pleurisy, with false membranes and adhesions; but experience has demonstrated that if the lung be bound down by false membranes, although very difficult, it is not impossible for it to assume its normal volume. On the other hand, the thoracic walls become depressed, the diaphragm elevated, as we have demonstrated above, and the cavity occupied by the liquid disappears. Besides, is it necessary to empty entirely the chest? Evidently it is not. The evacuation of a certain quantity of liquid may be imperatively demanded by the threatened suffocation, and in admitting that all the liquid may not have been evacuated by the first puncture, is there not immediate and marked relief? If the liquid is not evacuated by an operation, the subject dannot be relieved except by its absorption, and it is hardly necessary to say that when we evacuate half or two-thirds of the liquid we may with greater facility obtain the absorption of that which remains, because the quantity is not so great, and also the state of the subject is greatly ameliorated; besides, it is easily understood that absorption is much easier, all things equal, when the respiration is normal, than when the subject is in a state of approaching asphyxia.

3. *The danger of evacuating too great a quantity of liquid.*—This objection appears to us to be of very little value. What are the dangers that may result from the evacuation of a large quantity of liquid? Syncope? Has this accident been observed? Fears are yet entertained of the rupture of false membranes of long standing, but have we not seen above that the lung becomes distended with air only in proportion to the removal of the obstacle to its distension? We should have no fears then of removing too great a quantity of liquid, as its evacuation is always in proportion to the retreat of the walls of the thorax and the distension of the lungs.

4. *The impossibility to break up old adhesions.*—We discussed this objection when we spoke of the evacuation of liquid contained in the chest. We will now add only a few words: When the adhesions are too great the lung regains but imperfectly its functions, but if the operation relieves the dyspnœa, is not the object accomplished? The subject respires without difficulty from the healthy lung, and is certainly in the most favorable condition to regain the functions of the compressed lung.

6. *Rupture of the Pulminary Cells.*—We will not stop here to discuss this objection, as it has been demonstrated anatomically that this lesion does not take place.

7. *Inflammation of the pleura and lung as a consequence of the operation.*—This is a more serious objection; but do we not know that the peritoneum may become inflamed as a consequence of the operation of paracentesis abdominis? and yet no one objects to this operation from such fears, although he may know that there is no serous membrane which inflames so readily when wounded as the peritoneum.

8. *Reproduction of the Liquid.*—The liquid is more frequently and more rapidly reproduced in the abdomen than in the chest, yet no one has even thought to make this a serious objection against the operation of paracentesis abdominis.

9. *The persistence of a Thoracic Fistula.*—But this is not to be feared except in purulent effusions, and then by proper precautions we may prevent the constant flow of liquid.

It may be seen that among all these objections there is not one which should cause us to reject the operation; in truth, if there are cases in which it can be of no advantage, in quite a number it is of great utility, and does not present in reality the dangers which have proscribed it for such a length of time.

We will not here speak of the indications for this operation, as they have been discussed by M. REQUIN in a chapter above alluded to, and by us when speaking of the effusion of blood. We will then speak alone of the operation, and the accidents which may arise during its performance.

Before describing the diverse methods and procedures by which this operation has been performed, it is necessary to speak of some of the preliminary considerations.

It is of considerable importance at what point we open the thorax. When the collection of liquid is circumscribed by adhesions, and there is a projection exterior, we should make the opening at a point corresponding to this projection. It is the place of *necessity.* Thus, in a case reported by M. CRUVEILHIER,* a fluctuating tumor was rapidly formed under the nipple, and being surrounded by an edematous circle, was for him a sufficient evidence of an existing perforation, and the operation was performed at this point.

Authors have reported great success in cases of this character. It appears, as has been said, that art has more chances of success when nature has attempted a spontaneous evacuation of the liquid.

But when there is no sub-tegumentary tumor communicating with the pleura, nor a penetrating wound of the chest, when, in fact, the effused fluid occupies the whole of the pleural sac, we should make the puncture sufficiently low, but at a point where there would be no risk of wounding the diaphragm or the arteries. This is the place of election.

This place of election has varied. Each intercostal space, from the fourth to the eleventh, in counting from above downwards, has been proposed. In general, it is the union of the posterior third of the contour of the chest with the two ante-

* Dictionnaire de Medecine et de Chirurgie en 15 volumes. Vol. XIII, p. 312.

rior thirds, and as near as possible the superior border of the rib below that the puncture should be made.

Again, in determining the place of election it is important to refer to some practical considerations. If the operation is performed when the patient is in the sitting posture or bent forward, the lower the intercostal space selected the better will be the chance of puncturing the pleura at the most pendent point; but when the patient is in the horizontal position the six last intercostal spaces are very nearly upon the same plane.

SABATIER, BOYEN, and PELLETAN made the opening between the third and fourth ribs on the left side, and between the fourth and fifth on the right, counting from below upwards; CHOPART and DESAULT between the second and third ribs on the left side and the third and fourth on the right.

At present, it is the third intercostal space on the left, and the fourth on the right side, that is generally preferred.

But let us seiect whichever space we may, it is not always easy to determine definitely the exact point. Thus, if the subject be lean, we may be able to count the ribs by the touch, but if there be considerable *embonpoint*, or edema or emphysema of the sub-cutaneous cellular tissue, it is with great difficulty, and sometimes impossible, to recognise the intercostal spaces. It has been advised, under such circumstances, to make the puncture six fingers breadth below the inferior angle of the scapular; (M. VELPEAU,) or better, after applying the hand of the subject upon the sternum, to make the incision upon a line with the elbow when thrown a little back.

But these divers ways of determining the point for the incision are by no means exact; they vary with the heighth of the thorax, the length of the arm or scapula, and with the elevation of the shoulder and ribs. The best way to determine the point for the puncture, under such circumstances, is to find the border of the false ribs, which we may readily do, then make the incision three fingers breadth above the inferior border on the right side and two and a half on the left.

*Mode of Operating.*—There are three modes of performing the operation of paracentesis thoracis. They are, first, cauterization; second, incision; third, puncture. We will discuss them successively.

1. *Cauterization.*—HYPPOCRATES opened the thorax by passing a red hot iron between the intercostal spaces. This operation, although attempted to be re-established by M. GOURAND, is at present, with good reason, completely abandoned.

2. *Incision.*—While the patient sits upon the edge of the bed, with the body slightly bent towards the healthy side, in order to enlarge the intercostal spaces, the surgeon with the left hand makes tense the skin, and with a straight bistoury makes an incision an inch and a half or two inches long parallel to the intercostal space. After the skin, cellular tissue, and subjacent muscular fibres have been thus divided, and the intercostal muscle exposed, it has been advised to incise it with caution, approaching as near as convenient the superior border of the rib situated below to obviate wounding the intercostal artery. But this precaution is not so indispensable as some would have us suppose—the artery resting in a groove in the inferior border of the rib, which is generally sufficient to protect it.

When the pleura is exposed, we may, with the index finger of the left hand, feel the fluctuation of the liquid. We should then make an incision into the pleura, and the contents will be discharged.

M. VELPEAU, with the bistoury in the second or third position, plunges it directly into the pleural cavity, and enlarges the incision as he withdraws the instrument.

The mechanism of the evacuation of the liquid in the operation by incision is very easy to understand. The retreat of the thoracic walls, and the elevation of the diaphragm, expels a certain amount of liquid; in the movement of inspiration a certain amount of air from the exterior enters the cavity of the thorax, and at each respiratory movement a new quantity of liquid is discharged, which is replaced by a new quantity of air.

This method has with reason been abandoned, first, because it does not permit so readily the entry of air into the lung through the trachea, the lung does not become distended by the respiratory movement, as the pressure exercised by the liquid is replaced by the entry of air into the cavity of the chest; secondly, the subject is exposed to all the accidents that may arise from the introduction of air into the plural cavity. We will not again speak of this accident as we discussed it in an article under the head of *Penetrating Wounds of the Chest, with the effusion of blood.*

3. *Puncture.*—This operation is performed with an ordinary trocar, which is plunged directly into the chest, either at the place of *election* or at the place of *necessity*. The canula should be left after stopping it with a bit of cork to prevent the complete evacuation of the liquid, as well as to prevent the entry of air into the pleural cavity.

In leaving the canula thus with no support but the soft parts, it is subject to considerable motion, and may escape from the wound; again, the liquid may pass between the canula and the soft parts; for this reason M. REYBARD has proposed to perforate one of the ribs, and insert a metallic canula or a goose quill. To this method we greatly prefer that of M. TROUSSEAU, and of the ordinary trocar that of M. REYBARD.

The canula of M. REYBARD is nothing more than the canula of an ordinary trocar, with a sort of mesh or large, soft, and flexible tube, made of gold beater's skin or the intestines of a cat, or even a piece of bladder. This covering being previously wet does not prevent the evacuation of the liquid.

But if the air during the inspiratory movement tends to enter the canula, the softened tube or covering becomes flat and forms a sort of valve, which prevents its entry.

M. TROUSSEAU's mode of performing the operation is the following: He makes an incision through the skin with a lancet, just large enough for the trocar, and on a line with the inferior border of the eighth rib. An assistant now draws the skin upwards until the wound in the integuments corresponds with the seventh intercostal space. Then the surgeon places the index finger of the left hand upon the superior border of the eighth rib, and introduces the trocar into the incision by resting it on this finger. It is in raising the superior border of the eighth rib that the puncture is made through the intercostal muscle and pleura. When the trochar has entered the cavity of the pleura the blade should be withdrawn, having great care to adjust accurately the membrane upon the handle. The liquid is thus evacuated, and while it is being evacuated, we see the movements of the membranous valve. During expiration the membrane is raised; during inspiration, on the contrary, the liquid ceases to flow, and the membrane or valve is accurately applied to the orifice in the canula.

When the necessary amount of liquid has been evacuated, or when it ceases to flow, the canula, while the respiratory movements are suspended for a time, is rapidly withdrawn, the integuments assume their normal place, the parallelism between the incision in the skin and the puncture of the muscle and pleura is lost, and thus the air is prevented from entering the cavity of the chest.

From the commencement of the flow of the liquid, the surgeon or an aid should make pressure upon the abdomen to elevate the diaphragm, and thus favor the flow; the thorax should also be compressed to diminish the capacity of the pleural cavity; at the same time the patient should be requested to cough, and to make an effort as if he was at stool.

When a certain quantity of liquid has been discharged, the air enters the lung; the impression of air upon this organ, which has not respired for a long time, provokes a cough; at each effort of expiration the evacuation of a new quantity of liquid is effected, the lung yielding in proportion to the quantity of liquid evacuated. This distension of the lung is necessary to fill up the vacuum that the evacuation of the liquid tends to produce in the pleural cavity.

The return of the lung to its normal state produces in some cases the most violent pain. One of our honorable confriers, M. DRACHE, upon whom M. TROUSSEAU performed this operation, told us that there was nothing to compare to the pain that he experienced when, from the discharge of the liquid, the lung became distended and displaced.

After what we have said, it may be understood that the pain and cough consecutive to the operation should be considered as a happy prognostic, indicating that the lung is not bound down by false membranes and adhesions.

The accidents that may arise from paracentesis thoracis are:

1. The introduction of air into the pleural cavity. This accident which above all appears in the operation by incision, is extremely rare in the operation by puncture, with the precautions that we have indicated.

2. The injury of the lung by the extremity of the canula. The frequency and importance of this accident has been greatly exaggerated; it occurs after a fit of coughing, the lung during the inspiratory movement becoming distended with great rapidity. It is said to have produced pneumonia and also to have ruptured the pulmonary cells.

When the operation by puncture has been performed it sometimes becomes necessary to make injections. These injections have for aim, first, when there are clots and debris of pseudomembranes which prevent the free evacuation of liquid, they are used to dissolve these solids, that the liquid may more readily pass through the canula.

Again, injections are made to act upon the surface of the pleura as resolutives, substances to modify, excitants, and even caustics have been injected. The degree of irritation will determine our choice of the preparation to be used. Various substances have been proposed, as orange water with honey, aromatic infusions, a decoction of quinquina, and a weak solution of the nitrate of silver, or better, the injection of iodine, which has been used several times by M. Aran with success. Observation proves not only the harmlessness of these injections, but their great utility.

